# The future of genomics in Ireland – focus on genomics for health

**DOI:** 10.12688/hrbopenres.13187.1

**Published:** 2020-12-04

**Authors:** Cathal Seoighe, Adrian P. Bracken, Patrick Buckley, Peter Doran, Robert Green, Sandra Healy, David Kavanagh, Elaine Kenny, Mark Lawler, Maeve Lowery, Derek Morris, Darrin Morrissey, James J. O'Byrne, Denis Shields, Owen Smith, Charles A. Steward, Brian Sweeney, Walter Kolch

**Affiliations:** 1 National University of Ireland Galway, Galway, H91 TK33, Ireland; 2 Trinity College Dublin, Dublin, 2, Ireland; 3 Genuity Science (Ireland) Ltd., Dublin, D18 K7W4, Ireland; 4 University College Dublin, Dublin, 4, Ireland; 5 Mater Misericordiae University Hospital, Dublin, 7, Ireland; 6 Brigham Health, Broad Institute, Ariadne Labs, Harvard Medical School, Boston, MA, 02115, USA; 7 ELDA Biotech, Trinity Translational Medicine Institute, St James's Hospital, Dublin, D08 W9RT, Ireland; 8 Queen's University Belfast, Belfast, Northern Ireland, BT7 1NN, Ireland; 9 Saint James' Hospital, Dublin, D08 NHY1, Ireland; 10 National Institute for Bioprocessing Research and Training, Blackrock, A94 X099, Ireland; 11 Children’s Health Ireland, Crumlin, Dublin, D12 N512, Ireland; 12 Congenica, Wellcome Genome Campus, Hinxton, Cambridge, CB10 1DR, UK; 13 University College Cork, Cork, T12 YN60, Ireland

**Keywords:** Genomics, precision/personalized medicine, national genomics strategy, genome research, ethics of genome research, patient involvement, societal and economic aspects of genome research, health-economic assessment of clinical genomics

## Abstract

Genomics is revolutionizing biomedical research, medicine and healthcare globally in academic, public and industry sectors alike. Concrete examples around the world show that huge benefits for patients, society and economy can be accrued through effective and responsible genomic research and clinical applications. Unfortunately, Ireland has fallen behind and needs to act now in order to catch up. Here, we identify key issues that have resulted in Ireland lagging behind, describe how genomics can benefit Ireland and its people and outline the measures needed to make genomics work for Ireland and Irish patients. There is now an urgent need for a national genomics strategy that enables an effective, collaborative, responsible, well-regulated, and patient centred environment where genome research and clinical genomics can thrive.  We present eight recommendations that could be the pillars of a national genomics health strategy.

## Disclaimer

The views expressed in this article are those of the author(s). Publication in HRB Open Research does not imply endorsement by the Health Research Board of Ireland.

## Why have this discussion now?

Over the last decade, genomics and in particular the ability to sequence whole genomes rapidly and cheaply has changed our world dramatically and forever. While genomics has many applications, this paper focuses on genomics in biomedical research and clinical practice. There are over 7,500 genetic diseases (see Online Mendelian Inheritance in Man), which collectively are responsible for significant morbidity and mortality. The global prevalence of genetic diseases at birth is ~2.5/100 and it is estimated that an underlying disorder with a significant genetic component is present in up to 70% of children who are admitted to hospital ( McCandless *et al.*, 2004). A large proportion of genetic disorders are also diagnosed in adulthood ( Lynch *et al.*, 2019; Oosterloo *et al.*, 2019; Pfliegler *et al.*, 2014). Genomics can also provide significant insight to the biology of more complex diseases caused by a combination of genetic and environmental factors and has transformed both biomedical research and clinical applications, paving the way to personalised or precision medicine. It takes approximately 15–17 years to develop a new treatment, from initial discovery science to drug registration, but it took less than 10 years to bring genome sequencing from a pioneering worldwide research project (The Human Genome Project) to a valuable clinical diagnostic tool. Now, genomics is a robust technology with rapidly expanding applications in healthcare and across multiple industries as well as in scientific research. The Obama administration estimated that genomics had generated more than $1 trillion in economic benefits just in the first 10 years after the draft of the first human genome sequence was published in 2003 (see 'The Impact of Genomics on the U.S. Economy'). In the face of rapidly accelerating and expanding opportunities, can Ireland afford to miss out on this genomic revolution?

## What is the current state of genomics in Ireland?

Although clinical genetics/genomics is an established medical subspeciality in the Irish Healthcare system and genetics is a traditional research discipline in Irish Universities, Ireland has fallen behind in both research and clinical application of modern genomics compared to the USA, China, Japan and many other European countries ( Stark *et al.*, 2019). This gap is widening rapidly due to the fragmented structure of genome research in Ireland, lack of coordination between clinical and research genomics, and a lack of genomics infrastructure and capacity within the Irish healthcare system. The lack of resources is highlighted when comparing the number of clinical geneticists in Ireland to Northern Ireland. As of 2020, the Republic of Ireland has only 8 doctors registered on the specialist registrar for clinical genetics, i.e. 1:~700,000 population, whereas Northern Ireland has a ratio of 1:285,000 (see Health Service Executive review of the clinical genetics workforce). As clinical applications of genomics are highly dependent on the genomics research community as an innovation and implementation driver, the structural weakness in the Irish system negatively affects both research and healthcare systems ( Lynch & Borg, 2016). In combination with the lack of a national genomics strategy and associated implementation policies, this weakness has a particularly stifling impact on making clinical genomic services available to Irish patients. This affects both services provided by the government bodies as well as by commercial entities. Other countries have established clinical genome sequencing services either through public and academic facilities (e.g. Genomics England, Edinburgh Genomics, SciLifeLab, Broad Institute) or commercial entities (e.g. Q2 Solutions, Novogene, Foundation Medicine, Genewiz, Psomagen, Illumina). In Ireland the model remains undefined, especially the role of commercial entities active in genomics research and their relationship to the public research and healthcare sectors. Policies and guidelines are urgently needed to shape a coherent and productive genomics landscape in Ireland that will benefit healthcare, health-related research and especially Irish patients. The recent coronavirus disease 2019 (COVID-19) pandemic has vividly demonstrated that such shortcomings in regulation, policies, and research infrastructure can compromise rapid and efficient responses to such emergencies. The availability of genomics infrastructures and a functioning framework for genomic research in countries such as Australia and the UK has enabled powerful approaches for contact tracing, studying virus spread, and finding sources of infection ( Rockett *et al.*, 2020) (see UK Government press release on COVID-19 Genomics UK Consortium).

## How can genomics benefit Ireland?

***Health***. The patient population with a molecular diagnosis of genetic conditions or conditions with a genetic component is growing rapidly and will likely continue to increase for many years to come. This is due to 1) increased recognition and diagnosis rate of genetic conditions; and 2) medical advances in treatments which have resulted in increased survival of people with genetic conditions previously considered fatal, such as severe osteogenesis imperfecta, lysosomal storage disorders and mitochondrial disorders. Duchenne muscular dystrophy until recently was associated with inevitable death in the second decade but now, with improved treatment, survival rates have been expanded into the third and fourth decade ( Moxley *et al.*, 2010; Strehle & Straub, 2015). Successful gene therapy is now available and licenced for some genetic retinal dystrophies, which often present in adulthood, thus helping to prevent sight loss ( Dias *et al.*, 2018). Precision therapeutics are emerging and changing the patient journey for seizure disorders ( Kearney *et al.*, 2019). Genomics has already demonstrated that it can contribute to improved patient outcomes and, in certain diseases, to cost reduction. While concern at escalating costs has slowed its introduction into mainstream clinical services, there are now robust data emerging that genomics consistently reduces healthcare costs across different clinical applications ( Pennell *et al.*, 2019; Sabatini *et al.*, 2016; Shen *et al.*, 2015; Stark *et al.*, 2019; Stark *et al.*, 2017; Williams *et al.*, 2018), including in the Irish context ( Forman *et al.*, 2018). The savings arise from a number of areas including avoiding expensive sequential “single gene” testing, restricting the use of expensive but ineffective treatments in patients whose genetic make-up precludes a positive treatment response, and using genomics to stratify patients for better treatment outcomes, resulting in shorter hospital stays. For example, cancer is now the number one killer in Ireland; as of this year (2020) every second Irish citizen will get cancer, and that rate will further increase ( Dagenais *et al.*, 2020) and (see National Cancer Registry Ireland). Genomics has been proven to contribute to improved outcomes for cancer patients, while reducing costs by 50% or more ( Doble *et al.*, 2017; Gong *et al.*, 2018; Pennell *et al.*, 2019). However, the impact of genomics is much broader and similar cost-benefit results are seen in rare diseases and hereditary paediatric disorders ( Forman *et al.*, 2018; Sabatini *et al.*, 2016; Shen *et al.*, 2015; Stark *et al.*, 2019; Stark *et al.*, 2017). Genomics, molecular pathology, and analysis of the resulting disease characteristics are pre-requisites for incorporating precision medicine approaches into treatment strategies. Initial evidence also suggests that acceleration of diagnosis and shorter hospital stays can reduce waiting lists ( National Academies of Sciences *et al.*, 2018; Wurcel *et al.*, 2019). As an advanced knowledge-based society, we owe it to our patients and to the public to leverage and utilize the enormous possibilities of genomics to improve health and to reduce costs and waiting lists.

***Society***. Like many other EU countries, Ireland is struggling to cope with an ageing population and the impact of age-related diseases on society. In the future, we will have four generations living in parallel. Genomics will raise awareness of genetic heritage, genetic risk of disease, and also how genomics can be deployed to help treat disease. The Irish population is genetically relatively homogenous ( Byrne *et al.*, 2018; Gilbert *et al.*, 2017) and well suited for genetic research studies as well as for benefitting from the introduction of genomics into clinical care. Specific disease-causing genetic variants are often hidden in a sea of natural variations stemming from human population diversity. The larger and more homogenous a population is, the easier the task of identifying the disease-causing variants becomes. Thus, the “Irish Genome” should be regarded as a national and public resource, but value to Irish society will only ensue from using this resource in a responsible, informative and effective way ( Lawler *et al.*, 2018; Plun-Favreau *et al.*, 2016). Furthermore, the value of genomics can only be fully realised when genetic data are combined with rich clinical and other data related to the individual. Thus, genomics is a key cog in a citizen focussed multi-stakeholder collaboration and requires a constructive dialogue to develop an equitable social partnership if we are to realise its value for all stakeholders at a national level and in a sustainable manner.

***Innovation and economy***. Genomics has underpinned a burgeoning biotech industry around the world. Costs of sequencing are expected to fall further, but there is a convergence on an economy of scale, where a few market leaders will emerge. Data analysis and accurate deep phenotyping is becoming the bottleneck and value-limiting aspect of genomics. The ability to provide functional genome analysis and integration with other data types is key to economic exploitation. The island of Ireland has valuable collections of clinical disease characteristics and strong expertise in the integration of functional genome data ( Duffy *et al.*, 2015; Halasz *et al.*, 2016; Lawler *et al.*, 2017; Santra *et al.*, 2019; Siu *et al.*, 2016; Zhernovkov *et al.*, 2019). Thus, Ireland could take a leadership position in the economic exploitation of genomics. This opportunity deserves very serious consideration when designing clinical implementations of genomics. The US has already generated more than $1 trillion in economic benefits from genomics (see 'The Impact of Genomics on the U.S. Economy'). Genomics and genome sequencing services have spawned a flourishing biotech scene worldwide (see Genetic Engineering & Biotechnology News article) and in Europe (see Labiotech.eu article) that is expected to grow to a global market worth between $10.5 and $12.5 billion by 2022 (see Genetic Engineering & Biotechnology News article). Ireland cannot afford to miss this opportunity.

***Research***. Given the size and scale of research activities in Ireland, a coordinated genomics research community is needed to realise the potential of genomics for driving the healthcare benefits and cost savings described above. In genomics, research and clinical/biotech applications form a virtuous circle where one drives the other. Ireland has pockets of internationally very competitive research that – when combined – can close the gap in implementing functional genomics research in healthcare and also can generate economic value by promoting clinical trial activity and pharmaceutical research.

## How can Ireland gain the benefits of genomics?

Despite having many essential ingredients for genomics in place, such as strengths in biomedical research and data analytics, as well as economic sectors that create a market for genomics, Ireland has fallen behind. In order to allow Ireland to catch up with other developed nations and participate and compete in the genomic revolution, the following measures are needed.

**Eight recommendations for the implementation and regulation of genomics as an accountable, cost effective and efficient enabler of healthcare and health research:**

***1) A national policy is needed*** to establish clinical genomic services that are fit for purpose and future-proof. Countries with exemplary genomic services, such as France, Finland and England, have implemented genomics into the healthcare system through a top-down approach. Such a policy should address i) coordination of academic, clinical and commercial research in genomics; ii) a position on incidental findings both in the research and clinical care setting; iii) guidance to ensure equal access to genomics across the Irish population; and iv) mandating appropriate plans for data sharing in the context of large-scale genomics projects in the private and public domains. While it is clear that the development of such policies is the remit of Government Departments, the broader genomics community across the different sectors involved (healthcare, industry, academia) in Ireland is available to offer assistance in informing such policies by providing relevant domain expertise and supporting data. A coherent approach on the policy level regulating these relationships between public and private sectors will be essential to design the best and most cost efficient ways of providing genome sequencing services to Irish patients.

***2)*** We propose the assembly of ***an independent National Genomics Advisory Committee*** composed of academics, clinicians, patient representatives, and relevant government departments to guide the development of genomics policies and regulations in Ireland.

***3) True quantification of cost – benefit of genomics***. As elaborated above, genomics has been reported to reduce costs in clinical applications where it has been implemented and evaluated ( Doble *et al.*, 2017; Gong *et al.*, 2018; Pennell *et al.*, 2019) ( Forman *et al.*, 2018; Sabatini *et al.*, 2016; Shen *et al.*, 2015; Stark *et al.*, 2019; Stark *et al.*, 2017). However, these assessments mostly look at the cost of genomics versus a narrow segment of current standard diagnostics, treatments and care. What has not been considered adequately so far, are savings resulting from genomics replacing a number of currently used tests. These savings could be substantial. For instance, the cost of a clinical exome sequence is similar to the cost of 3 single gene tests that are currently used for detecting mutations in cancer patients. Cost savings could be especially significant when genomics is used to select patients for expensive therapies where a treatment cycle costs in the range of €60,000 (e.g. Herceptin) - €100,000 (RAF inhibitors, immunotherapies). Only treating patients whose genomic make-up indicates that they will respond to a therapeutic agent will lead to significant cost efficiencies within treatment pathways.

***4) Alignment with and incorporation of genomics into Sláintecare***. Sláintecare is the proposed major initiative to reform the healthcare system in Ireland, putting the patient at the centre of the system and creating opportunities for better coordination of healthcare. Undoubtedly, clinical genomics should have a defined role and place in Sláintecare. We strongly believe that genomics can amplify the impact of Sláintecare for Irish patients, if incorporated as part of the clinical care pathway.

***5) Implementing genomics to underpin clinical trials***. Clinical trials are a key component of health research that create benefits for patients while saving cost (see Clinical Research Development Ireland report). Ireland is lagging behind in clinical trial activity compared to other EU countries with similar population sizes (see HRB review of Irish clinical infrastructure). Incorporating genomics as standard practice into Irish clinical research will provide robust data that will improve the quality of clinical trial investigations and contribute to the infrastructure required for increasing the number of clinical trials. A robust genomics platform in the Irish health system has the potential to more strongly embed clinical trials and clinical investigations into the Irish health system. This will help both patients and sponsors, because it provides a quicker understanding of why certain patients are benefitting, and why certain patients should avoid a particular drug treatment (in some cases explaining serious adverse events). Currently, Irish clinical trial activities mainly rely on charitable organisations, such as Cancer Trials Ireland, and contract research organisations (CROs), such as ICON and IQVIA. Ireland currently takes very limited advantage of the strong presence of major pharmaceutical companies on its shores, but an investment in genomics would put Ireland in a much stronger position when it comes to companies considering where to locate clinical trials.

***6) Investment in infrastructure for clinical genomics***. Currently, this investment is fragmented, too small and consequently inefficient. In particular, for healthcare applications, centralised services with integrated data generation, and secure cloud-based data management and analysis can provide the required high quality, robustness and economy of scale that keeps them sustainable and expandable. The appointment of a new Professor of Genomics between University College Dublin (UCD) and Trinity College Dublin (TCD) is a valuable first step into this direction but needs to be supported by collateral diagnostics and therapeutics programmes.

***7) Investment in education and training***. Genomics is a biomedical data science and clinical genomics is the integration of genomic information into healthcare. Despite a worldwide, fast-growing demand, there remains a significant shortage of doctors trained as clinical geneticists/genomicists, subspecialist doctors with an interest in the genomics as their area of expertise, genetic counsellors, bioinformaticians and analytical data scientists. In order for Ireland to partake in this expanding sector of the knowledge industry, a coordinated nationwide Genomic Education Programme is needed. The SFI Centres for Research Training programme is a step in this direction but should be complemented by bespoke training programmes at both educational and professional levels.

***8) Recognising the patient as the centre of the genomic revolution: implementing a nationwide public engagement and involvement programme for genomics***. Genomics has raised huge public interest from ethical, societal and privacy perspectives. The ultimate success of the integration of genomics will rely on the skills of the professionals in our universities and health system, but more importantly on the co-operation and contributions of patients and the public to shape and facilitate this important research. Healthcare is about improving patients’ lives. With the prospect of making treatments personalised, genomics emphasizes that the patient needs to be an active participant and protagonist in shaping the aims and implementation of genomic research and clinical services in Ireland. Recognising the lead role of patients, we need to design mechanisms that place them at the heart of the ecosystem. We, therefore, advocate a nationwide public engagement and involvement programme that brings all stakeholders together, that provides objective, unbiased information and openly debates how to integrate genomics, including commercial partners, into the public healthcare and research systems. The result of such engagement should be a “social contract” of trust, which assures that genomics data provided by members of the public will be used responsibly and for the wider good ( Lawler *et al.*, 2018).

## The way forward: Developing an integrated model for genomics in Ireland

This document articulates the views of experts on the current state and future of genomics in Ireland, including representatives of the research and clinical community, patient advocates, funders and industry in Ireland as well as international thought leaders. Our aim is to initiate a discussion between stakeholders that is inclusive and can reconcile competing interests and objectives. The healthcare question to be solved is how best to incorporate genomics into the current Irish healthcare ecosystem in a manner that balances benefits and cost. The economic question to be solved is the relationship between healthcare, academic-led research and industry-led research. The societal question is how we best structure these relationships to optimise the benefits for the citizens of Ireland and assure equal access to the genomics services that provide these benefits. With this document, we reach out to the Irish Government to offer a proactive engagement and our domain-specific expertise for structuring and implementing clinical genomics and genomic research in Ireland.

## Data availability

### Underlying data

No data are associated with this article.
