# Supplementary material for: The future of genomics in Ireland – focus on genomics for health
Source: HRB Open Res. 2020 Dec 4;3:89. [Version 1] doi: 10.12688/hrbopenres.13187.1 (PMC7993626; doi:10.12688/hrbopenres.13187.1)
Supplement: Peer review history

# Peer review: Reviewer response for version 1

**DOI:** 10.21956/hrbopenres.14320.r29024

**Published:** 2021-03-23

**Authors:** Gabrielle Bertier

**Affiliations:** 1 Imagia Cybernetics, Montreal, QC, Canada

This article describes the state of the field of genomics in Ireland, with a focus on healthcare. After listing current limitations, the authors provide concrete recommendations to better integrate and support genomics for the benefit of patients in Ireland.

 The letter is clear, concise, well documented and provides concrete, reasonable recommendations. It would be interesting to add slightly more details on current sequencing infrastructures in Ireland (presence of ISO certified genomic laboratories and their capacities). Similarly, having an understanding of Irish patients' current access to genomic testing (e.g. the number or proportions of cancer patients who get clinical WES every year) could be helpful to the reader. One domain that is not mentioned in the article is prenatal screening, and Non Invasive Prenatal Testing technologies - it could be relevant to add a few words on this topic. It would also be critical to mention Genomics Medicine Ireland and how that impacts the national strategy for clinical genomics.

 Finally, two statements in the article would benefit from further explanation of detail:

1. "The recent coronavirus disease 2019 (COVID-19) pandemic has vividly demonstrated that such shortcomings in regulation, policies, and research infrastructure can compromise rapid and efficient responses to such emergencies."  What are these shortcomings in Ireland specifically, and how has this impacted the COVID-19 response?
2. "Other countries have established clinical genome sequencing services either through public and academic facilities (e.g. Genomics England, Edinburgh Genomics, SciLifeLab, Broad Institute) or commercial entities (e.g. Q2 Solutions, Novogene, Foundation Medicine, Genewiz, Psomagen, Illumina)". More than listing the commercial entities in the second part of the sentence, it would be helpful to place these in the context of specific national strategies. 

Does the article adequately reference differing views and opinions?

Partly

Are all factual statements correct, and are statements and arguments made adequately supported by citations?

Partly

Is the Open Letter written in accessible language?

Yes

Where applicable, are recommendations and next steps explained clearly for others to follow?

Yes

Is the rationale for the Open Letter provided in sufficient detail?

Yes

Reviewer Expertise:

Ethical, Legal and Social Issues of Genomics, Clinical genomics, national genomic policies, oncology and rare diseases.

I confirm that I have read this submission and believe that I have an appropriate level of expertise to confirm that it is of an acceptable scientific standard.

# Peer review: Reviewer response for version 1

**DOI:** 10.21956/hrbopenres.14320.r29028

**Published:** 2021-03-15

**Authors:** Edward Abrahams

**Affiliations:** 1 Personalized Medicine Coalition, Washington, DC, USA

I strongly recommend that “The future of genomics in Ireland” be indexed.

 Well-written and strongly argued, the article is compelling and persuasive. The case for integrating genomics and personalized medicine into the Irish health system, according to the authors, is clear. They show that patients can and will benefit from improved outcomes and that the Irish health system, if it is smart enough to embrace what they propose, will also derive increased efficiencies and therefore lower overall costs if it takes advantage the increased insights provided by the ongoing genomic revolution. Its “call to action” is well placed. 

 My only criticism of the article is that I think that the authors may exaggerate other nations’ embrace of genomics as the engine of innovation. Even in England, Finland, and the United States, all three of which are cited as reference points for the Irish experience, the commitment to using genomics to advance health care is still haphazard and typically confined to research.

 We have our work cut for us, but this article represents presents the case why it is worth doing.

Does the article adequately reference differing views and opinions?

Yes

Are all factual statements correct, and are statements and arguments made adequately supported by citations?

Yes

Is the Open Letter written in accessible language?

Yes

Where applicable, are recommendations and next steps explained clearly for others to follow?

Yes

Is the rationale for the Open Letter provided in sufficient detail?

Yes

Reviewer Expertise:

NA

I confirm that I have read this submission and believe that I have an appropriate level of expertise to confirm that it is of an acceptable scientific standard.
